# Modulation of inflammatory mediators in the trigeminal ganglion by botulinum neurotoxin type A: an organ culture study

**DOI:** 10.1186/s10194-015-0555-z

**Published:** 2015-08-06

**Authors:** Jacob Edvinsson, Karin Warfvinge, Lars Edvinsson

**Affiliations:** Department of Medicine, Lund University, Lund, Sweden; Department of Clinical Experimental Research, Glostrup Research Institute, Glostrup Hospital, Glostrup, Denmark

**Keywords:** CGRP, iNOS, IL-1β, SNAP-25, SV2-A, Monoclonal antibodies, Botulinum neurotoxin type A

## Abstract

**Background:**

Onabotulinumtoxin type A (BoNT-A) has been found to reduce pain in chronic migraine. The aim of the present study was to ask if BoNT-A can interact directly on sensory mechanisms in the trigeminal ganglion (TG) using an organ culture method.

**Methods:**

To induce inflammation, rat TGs were incubated for 24 hrs with either the mitogen MEK1/2 inhibitor U0126, BoNT-A or NaCl. After this the TGs were prepared for immunohistochemistry. Sections of the TG were then incubated with primary antibodies against CGRP (neuronal transmitter), iNOS (inflammatory marker), IL-1β (Interleukin 1β), SNAP-25 (synaptic vesicle docking protein) or SV2-A (Botulinum toxin receptor element).

**Results:**

We report that CGRP, iNOS, IL-1β, SNAP-25 and SV2-A were observed in fresh TG with a differential distribution. Interestingly, NaCl organ culture of the TG resulted in enhanced expression of CGRP and SNAP-25 in neurons and iNOS in SGCs. Co-incubation with U0126 or BoNT-A retained the increased expression of SNAP-25, while it decreased the IL-1β immunoreactivity in neurons. The iNOS expression in SGCs returned to levels observed in fresh specimens. Moreover, we observed no alteration SV2-A expression in SGCs. Thus, the overall picture is that both U0126 and BoNT-A have the ability to modify the expression of certain molecules in the TG.

**Conclusion:**

We hypothesize that chronic migraine might be associated with some degree of inflammation in the TG that could involve both neurons and SGCs. It is clinically well recognized that treatment with corticosteroids will reduce the symptoms of chronic migraine; however this remedy is associated with long-term side effects. Understanding the mechanisms involved in the expressional alterations may suggest novel ways to modify the changes and indicate novel therapeutics. The results of the present work illustrate one way by which BoNT-A may modify these expressional alterations.

## Background

Migraine is a common neurological disorder that afflicts up to 16 % of the adult population in the Western countries [1]. It is characterized by episodic, often disabling headache, associated with sensory, autonomic, central nervous system (CNS) related and cognitive symptoms. The current view is that migraine is a disorder in which CNS dysfunction plays a pivotal role while various parts of the trigeminal system are necessary for the expression of peripheral symptoms and aspects of pain [2].

In a subgroup of migraine patients (1–2 %) the frequency of migraine may expand over time to multiple monthly attacks. Furthermore, these may progress to be chronic (attacks > 15 days per month) and are often associated with medication overuse [3]. These patients are very difficult to treat. Onabotulinumtoxin type A (BoNT-A) has shown efficacy in the treatment of chronic migraine [4–6], however its mechanism of action remains in this relation elusive.

The effect of BoNT-A at the neuromuscular junction is well demonstrated [7]. The C-terminal of the toxin binds to the motor neuron and mediates endocytosis of the toxin [8]. Within the vesicle of the motor endplate it cleaves the vesicular docking protein SNAP-25, which leads to inhibition of acetylcholine storing vesicles docking on the presynaptic membrane and thus reduces acetylcholine release [8]. The potential of BoNT-A in treatment of migraine was suggested 15 years ago and observed in conjunction with cosmetic treatments [9]. Since then several suggestions to explain the antimigraine effect has appeared. The most obvious would be reduction in proprioceptive signaling to the brainstem but also decreased mechanical sensitivity of nociceptors and inhibition of craniofacial muscle tone [10–12]. These mechanisms were further developed and BoNT-A was suggested to interfere with expression of mechanosensitive ion channels on meningeal nociceptors [13].

The aim of the present study was designed to ask if BoNT-A can interact directly on sensory mechanisms in the trigeminal ganglion (TG) using an organ culture method [14–16]. With this method we can study whole TG, and the interrelation between neurons and satellite glial cells (SGC). The neuronal-glial signalling in the TG might be of much relevance in migraine and in particular in the chronification pathology that develops in many patients [17]. During organ culture there is an inflammation response elicited with increased expression of cytokines and mitogen-activated protein kinases (MAPK) [14–16]. We hypothesized that BoNT-A might interfere with the expressional changes of the induced inflammation, and, in addition, the expression of SNAP-25 and the Botulinum toxin receptor element (SV2), molecules observed in the TG.

## Methods

Ten Sprague Dawley rats (male, 200–250 g) were anesthetized with CO_2_ and decapitated. Right and left TG were removed and either used directly for experiments (fresh), or incubated in Dulbecco’s modified Eagle’s medium (DMEM; Gibco, Invitrogen, Carlsbad, CA, USA) supplemented with penicillin (100 U ml^−1^), streptomycin (100 μg mL^−1^) and amphotericin B (0.25 μg mL^−1^) for 24 hours at 37 °C in humidified 5 % CO_2_ in air (for details on the method see Tajti et al.) [15]. Prior to start of the incubation the MEK1/2 inhibitor U0126 (LC laboratories, Boston, MA, USA) 10 μM, BoNT-A (3 units/mL, ALLERGAN) or an equal volume of NaCl (vehicle) was added. Incubation with the different substances was repeated 5–7 times. The experimental procedures were approved by the University Animal Ethics Commtttee (M43-07).

TG (either fresh or after 24 hours of incubation) were fixated in 4 % paraformaldehyde (Sigma, St Louis, USA) in phosphate buffered saline (PBS) for 2–4 hours. After fixation TG were cryoprotected using 10 % and 25 % sucrose (Sigma) in Sorensen’s phosphate buffer. Subsequently, the specimens were embedded in gelatin medium (30 % egg albumin, 3 % gelatin, Sigma), cryosectioned at 12 μm and stored at −20 °C until use.

Sections were thawed in room temperature, then rehydrated in PBS containing 0.25 % Triton X-100 (PBS-T; Sigma) for 15 minutes. Sections were incubated with primary antibodies in PBS-T, containing 1 % bovine serum albumin (BSA; Sigma), overnight in +4 °C. After incubation with the primary antibody, sections were equilibrated to room temperature, rinsed in PBS-T for 2×15 min, followed by incubation with the secondary antibody for 1 hour in a dark room at room temperature (for details on antibodies, see Table 1). Sections were washed with PBST 2×15 min and mounted with anti-fading mounting medium (Vectashield, Vector Laboratories, Burlingame, CA, USA). Omission of the primary antibody served as negative control. The immune-stainings were repeated 3–5 times. For general morphology evaluation, sections were stained in hematoxylin-eosin (Htx, Sigma).

**Table 1:** Details on primary antibodies used for immunohistochemistry

Name	Host	Dilution	Supplier
iNOS	Rabbit	1:200	Abcam; Cambridge, UK
IL1β	Rabbit	1:400	Abcam; Cambridge, UK
SNAP-25	Rabbit	1:100	Sigma-Aldrich, St. Louis, MO, USA
SV2-A	Rabbit	1:1000	Abcam; Cambridge, UK

Sections were examined and images were obtained using light- and epifluorescence microscope (Nikon 80i, Tokyo, Japan) equipped with a scanning stage for upright microscope (Märzhäuser, Germany) with travel range X/Y 75 × 50 mm automatic adjustment of the Z-axis, and coupled to a Nikon DS-2 MV camera. This enabled us to take large images of TG, with high resolution. Images were taken using NIS basic research software (Nikon, Japan). We estimated the number of immunoreactive cells and the intensity in these images; however, the detailed distribution was analyzed in regular images in 20× and 40× magnification. In addition, as a complement to the analysis, fluorescence intensity was measured in three sections of CGRP, IL-1β and SNAP-25 stainings, since these showed neuronal immunoreactivity which could be measured. Mean intensity and SD were calculated. In iNOS and SV2-A stainings, the immunoreactivity was confined to the SGCs and could thereby not be used in fluorescence intensity measurements. Statistically, one-way ANOVA analysis and Bonferroni’s multiple comparison test were used. *P* < 0.05 was considered as statistically significant.

## Results

### Morphology

**Fig. 1:**
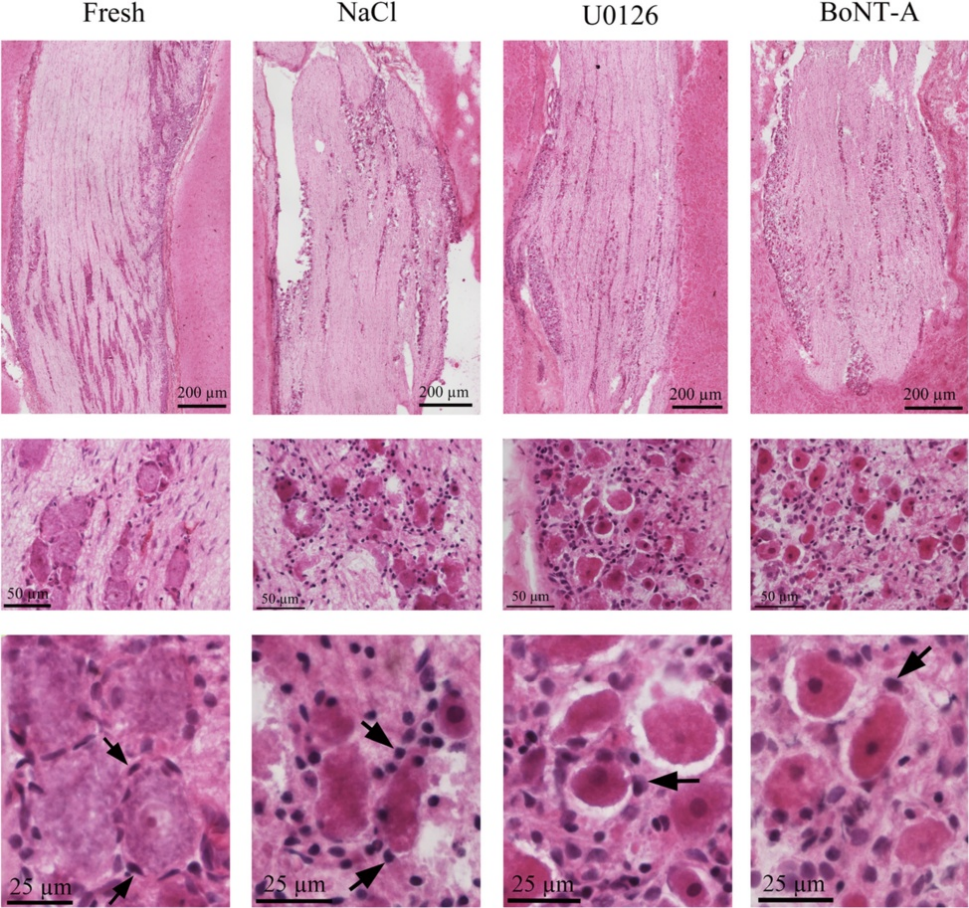
Hematoxylin-Eosin staining. The column to the left shows the trigeminal ganglion in a fresh rat. The neurons were firmly enveloped by the SGC (arrows), demonstrating the close interaction between the neurons and glial cells. In comparison to a neuron, which contained a large pale nucleus and a visible nucleolus, the SGCs displayed a slender, slightly condensed nucleus. In the specimens that underwent incubation during 24 hrs, a similar morphology between the three groups was found: neurons with both condensed cytoplasm and nuclei, and enlarged rounded SGCs with highly condensed nuclei (arrows). In groups incubated with U0126 or BoNT-A, the neurons were often found in a vacuole indicating SGC detachment

The morphology of the TG was evaluated following Htx staining (Fig. 1). Neurons of different size were firmly enveloped by SGCs, demonstrating the close interaction between the neurons and the glial cells. The neurons contained a large pale nucleus and a visible nucleolus, and the SGCs displayed a slender, slightly condensed nucleus.

In the specimens that underwent incubation during 24 hours, a similar morphology between the three groups was observed: neurons with both condensed cytoplasm and nuclei, and enlarged rounded SGCs with highly condensed nuclei. Moreover, in groups treated with U0126 or BoNT-A, the neurons were often found in a vacuole indicating cell shrinkage and SGC detachment.

### Fresh TG

**Fig. 2:**
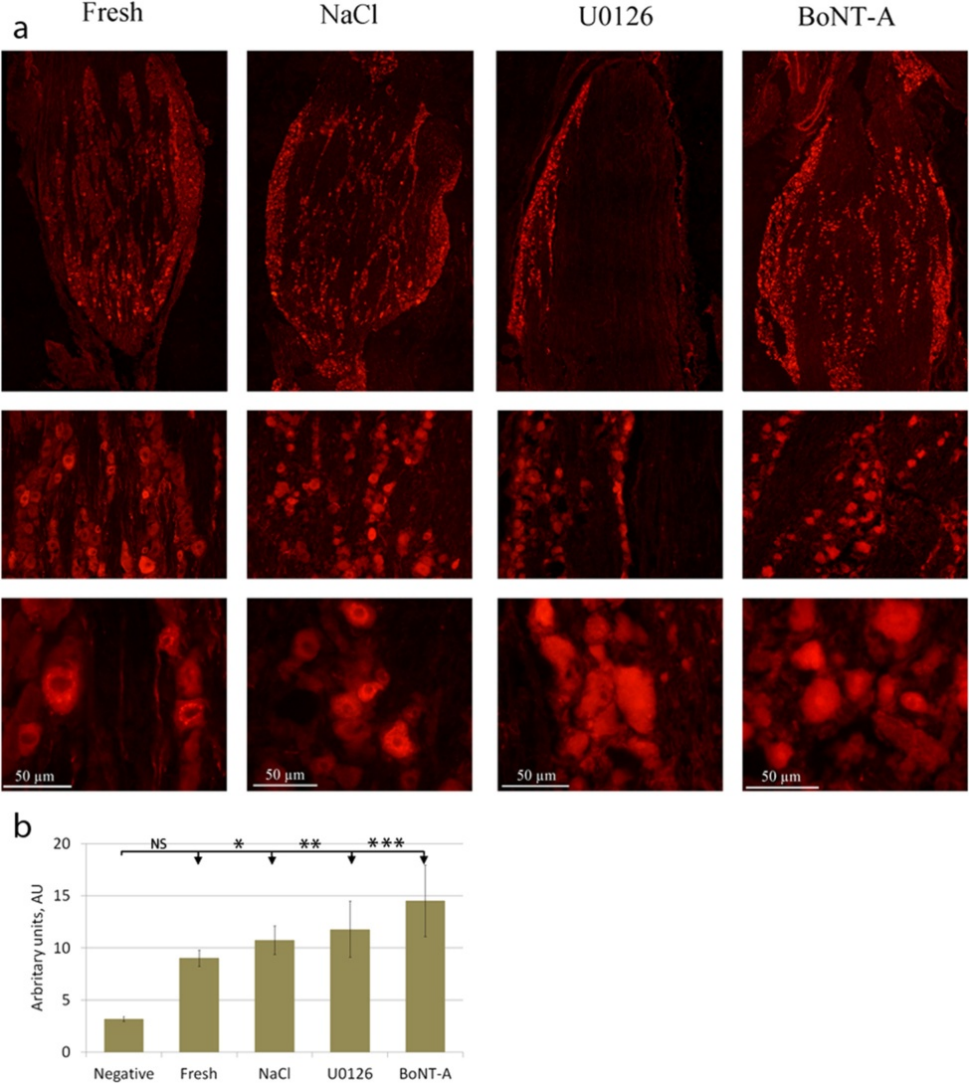
CGRP immunohistochemistry. **a** Fresh TG contained both CGRP positive and negative neurons. The immunoreactivity was confined to the cytoplasm in a granular pattern, resembling staining of the endoplasmatic reticulum, and in some of the fibers. In TG incubated in medium containing saline, the number of neurons immunoreactive to CGRP seemed to be increased. The immunoreactivity was often spread in a granular matter in the entire cytoplasm as observed in the fresh TG. In the specimens incubated with U0126 or BoNT-A, the number of positive neurons increased and the immunoreactivity was found in the entire cytoplasm in almost all neurons. **b** To illustrate the microscopical findings, fluorescence intensity measurements were performed. The results confirm these findings; a tendency of intensity increase after 24 hrs of incubation with NaCl, U0126 or BoNT-A. Negative column is the same as in SNAP-25 diagram since the same secondary antibody is used in both experiments. Bars indicate SD

In the fresh TG, we observed both CGRP positive and negative neurons (Fig. 2a). The CGRP immunoreactivity was confined to the cytoplasm in a granular pattern, resembling staining of the endoplasmatic reticulum. In addition, some of the nerve fibers seen in TG were CGRP positive.

**Fig. 3:**
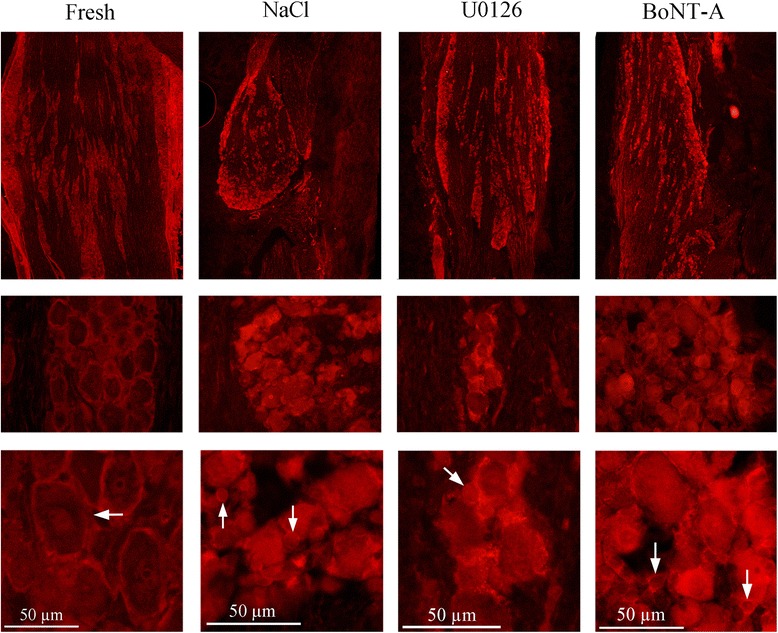
iNOS immunohistochemistry. In fresh animals, the iNOS immunoreactivity was exclusively found in the SGCs (arrow). In the saline group, some neurons and neuronal nuclei in addition to the SGCs (arrows) displayed immunoreactivity. In the groups incubated with U0126 or BoNT-A the immunoreactivity had returned to levels found in fresh TG

**Fig. 4:**
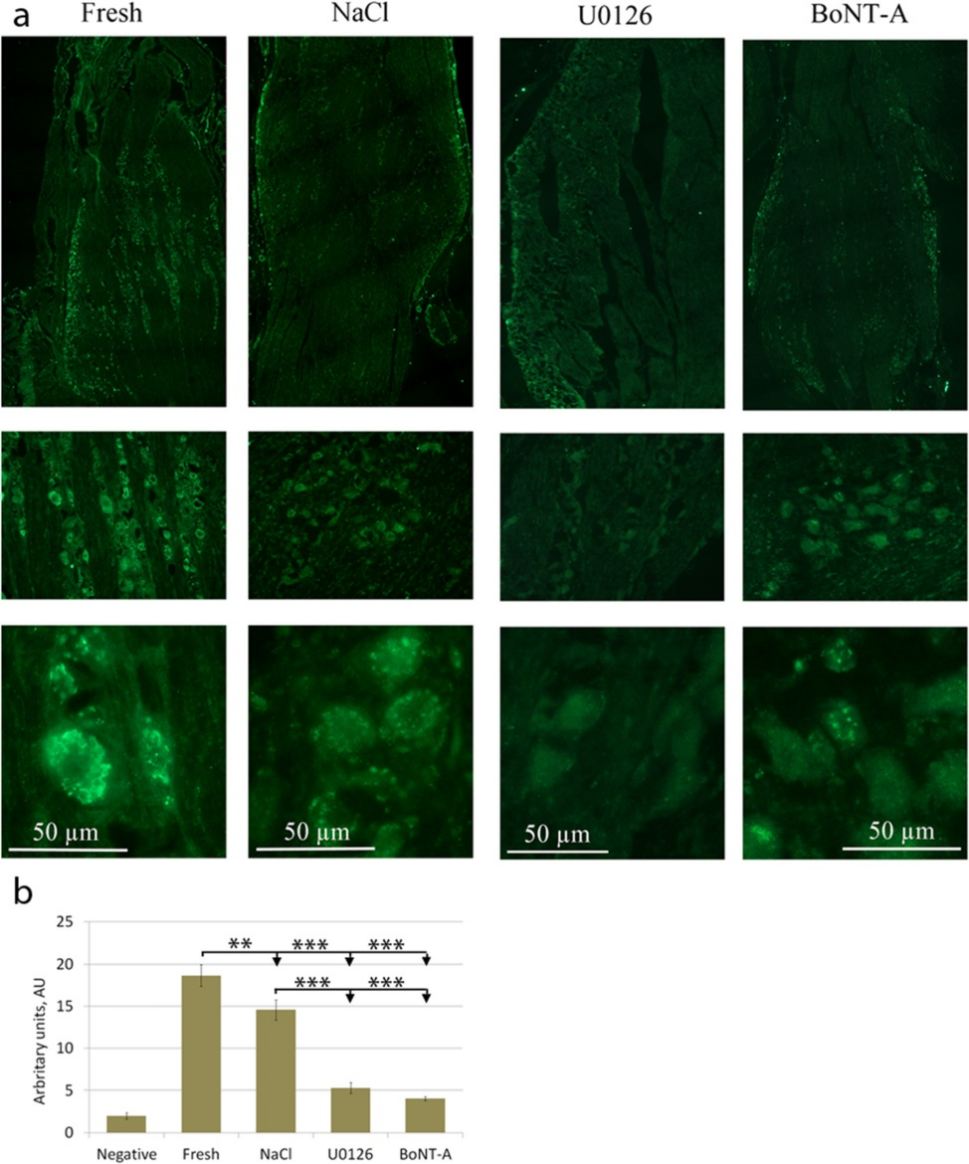
IL1β immunohistochemistry. **a** Almost all neurons in the fresh specimens displayed IL1β immunoreactivity, which appeared as granules in the cytoplasm. This pattern was also found in the saline incubated groups. The groups incubated with U0126 or BoNT-A showed none or little IL1β immunoreactivity. **b** Fluorescence intensity measurements confirmed these findings. Bars indicate SD

iNOS immunoreactivity was exclusively found in the SGCs (Fig. 3), and IL-1β immunoreactivity in almost all neurons, which appeared as granules in the cytoplasm (Fig. 4a).

**Fig. 5:**
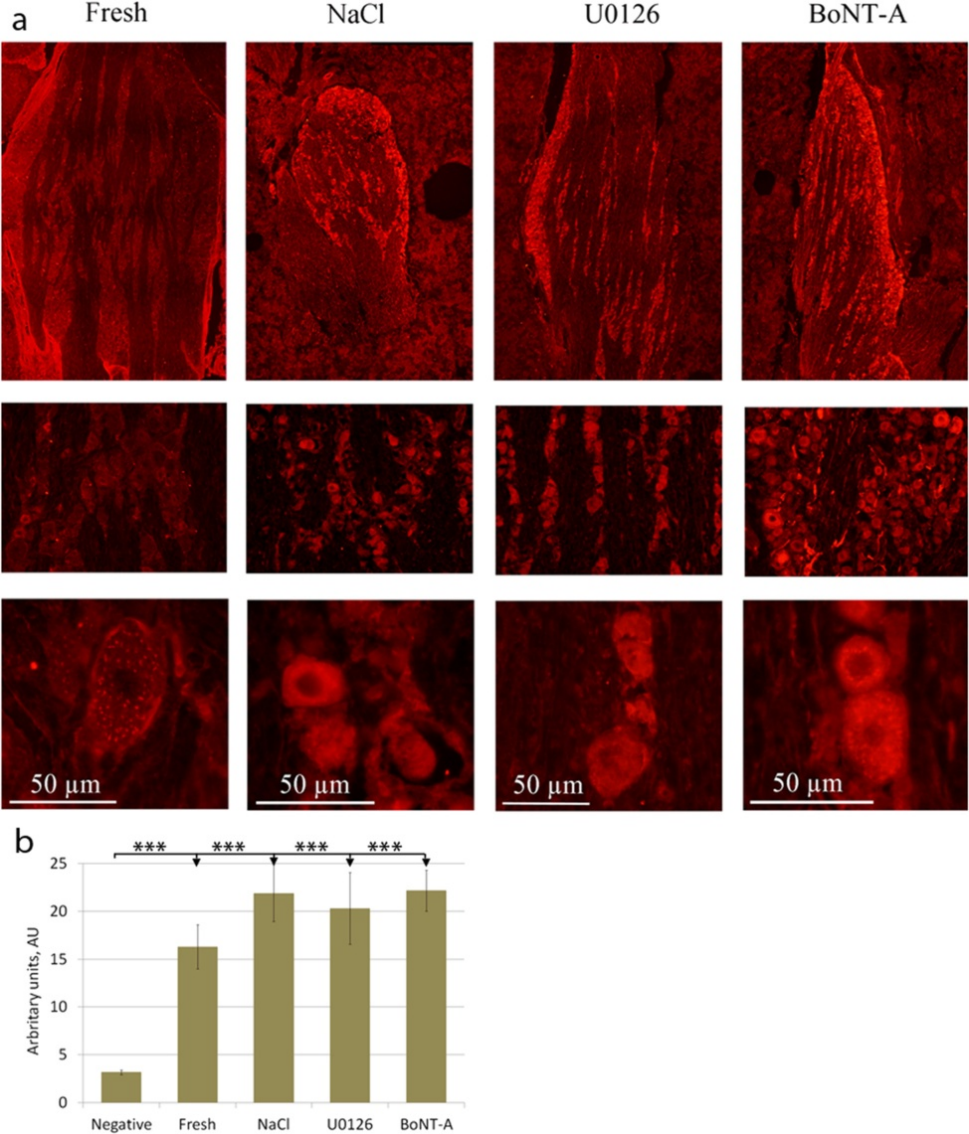
SNAP-25 immunohistochemistry. **a** Scattered cytoplasmatic SNAP-25 immunoreactive granules were found in most neurons in the fresh specimens. The intensity of the staining in the incubated groups increased intensively, making it difficult to discern the distribution of immunoreactivity within the neurons. **b** Fluorescence intensity measurements confirmed these findindings. Negative column is the same as in SNAP-25 diagram since the same secondary antibody is used in both experiments. Bars indicate SD

**Fig. 6:**
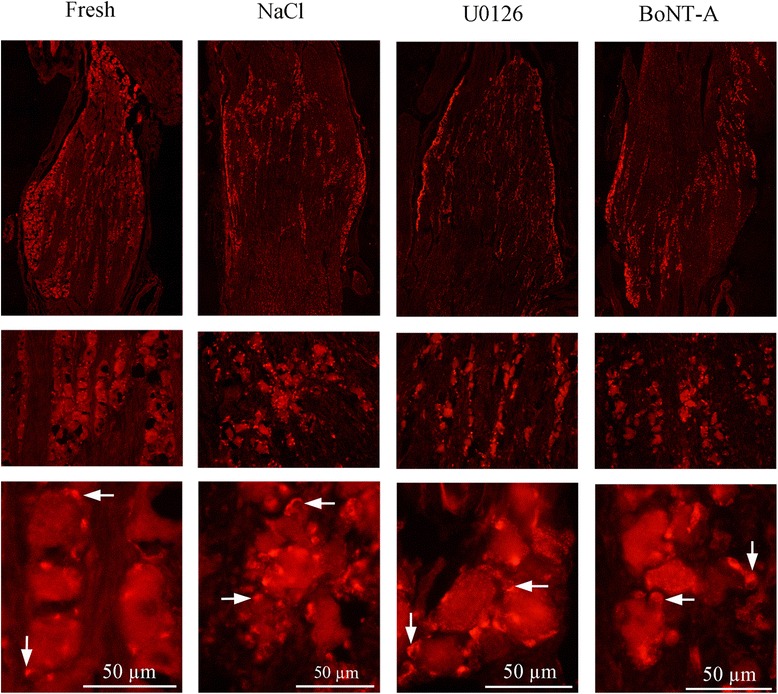
SV2-A immunohistochemistry. In all groups, including fresh, SV2-A immunoreactivity was confined to the cytoplasm of SGCs (arrows). No difference was detected between the groups, except for the rounded shape of the SGCs appearing after 24 hrs of incubation

Scattered cytoplasmatic SNAP-25 immunoreactive granules were found in most neurons (Fig. 5a) and SV2-A immunoreactivity in the cytoplasm of SGCs (Fig. 6).

### Vehicle incubation for 24 hrs

In the NaCl incubated group (vehicle group), we observed no clear difference in CGRP immunoreactivity compared to fresh TG; both CGRP immunoreactive and negative neurons were observed (Fig. 2a). In addition, the granular intracytoplasmatic CGRP immunoreactivity found in fresh TG was found after 24 hours of incubation with NaCl. Immunoreactive CGRP positive fibers were also observed.

The SCGs displayed increased iNOS immunoreactivity compared to fresh TG (Fig. 3). As for the fresh specimens, almost all neurons in the incubated groups displayed IL-1β immunoreactivity, which appeared as granules in the cytoplasm (Fig. 4a).

Compared to the fresh specimens, there was increased SNAP-25 immunoreactivity in the neurons in the NaCl incubated group, to such an extent that it was difficult to discern the distribution of immunoreactivity within the neurons (Fig. 5a). We found no difference in SV2-A immunoreactivity between the fresh specimens and the incubation group (Fig. 6), except for the rounded shape of the SGCs appearing after 24 hours of incubation, as also was seen in the Htx staining (Fig. 1).

### U0126 and BoNT-A incubation for 24 hrs

Compared to the fresh specimens, the number of CGRP positive cells was clearly increased in the U0126 group. This was more marked in the BoNT-A treated TG (Fig. 2a). In addition the distribution of the immunoreactivity differed compared to fresh and saline groups; the immunoreactivity was found in the entire cytoplasm in almost all neurons compared to the granular pattern observed in the other groups.

Compared to NaCl incubated group, iNOS immunoreactivity has returned to levels found in fresh TG (Fig. 3) and IL-1β immunoreactivity has decreased compared to fresh, or saline group (Fig. 4a).

The cytoplasmatic SNAP-25 immunoreactive granules found in neurons in the fresh specimens were visible again (after being hidden in the NaCl group by the high intensity of the staining), even though the intensity was considerably increased in the U0126 and BoNT-A groups compared to fresh (Fig. 5a). No difference could be seen in the SV2-A immunoreactivity between all the groups examined (Fig. 6).

Fluorescence intensity measurements of CGRP, IL-1β or SNAP-25 were performed on incubated specimens (Figs. 2b, 4b, 5b). The intensity in the neurons was measured and the mean intensity was calculated. Statistically, one-way ANOVA analysis and Bonferroni’s multiple comparison test were used. The measurements confirmed the microscopical results and are presented in the illustrations.

**Fig. 7:**
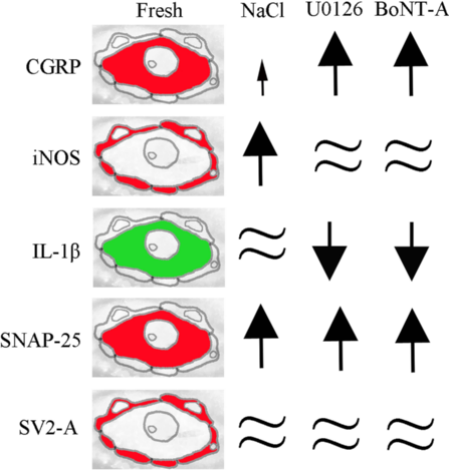
Schematic drawings. The drawing demonstrates the major immunohistochemical findings in neurons and SGCs. CGRP, IL1β and SNAP-25 were mainly found in the neurons, and iNOS and SV2-A in the SGCs. Arrows illustrate increase and decrease, respectively.

Major findings are illustrated schematically in Fig. 7.

## Discussion

The present study was designed to examine isolated TG neurons and SGCs during different organ culture conditions as a method to illustrate possible expressional alterations of CGRP, iNOS, IL-1 β, SNAP-25 and SV2-A. The underlying hypothesis is that chronic migraine is associated with inflammation in various parts of the trigeminal system and organ culture is a way to induce an inflammatory response in the TG. The advantage of this method is the study of neurons and SGCs in their normal habitat. We report that the studied molecules were observed in fresh TG with a differential distribution. Interestingly, organ culture co-incubated with the vehicle NaCl of the TG for 24 hours resulted in enhanced expression of CGRP and SNAP-25 in neurons, and iNOS in SGCs. Co-incubation with U0126 and BoNT-A retained the increased expression of SNAP-25, while it decreased the IL-1 β immunoreactivity in neurons. The iNOS expression in SGCs returned to levels observed in fresh specimens. Moreover, we observed no alteration of SV2-A expression in SGCs. However, the overall picture is that both U0126 and BoNT-A have the ability to modify/reduce the expression of certain molecules in the TG.

CGRP was used to provide further in depth information how and if this system are involved using our method. Organ culture results in increased expression of CGRP immunoreactivity in the TG neurons at 48 hours [14, 15], but only slightly at 24 hours (present data). Although we did not perform any detailed cell counting in the present study, the localization resembles that described by Eftekhari et al. (2010) [18].

iNOS is expressed by a wide variety of cell types, including neurons and SGCs [19]. We show that iNOS is expressed exclusively in SGCs of the TG. Increased iNOS expression in glial cells is implicated in the etiology of CNS diseases by causing inflammation and cytotoxicity [20, 21]. Inflammatory stimuli, in our case incubation for 24 hours with saline, revealed increased expression of iNOS in SGCs as compared to non-incubated TG. It has been demonstrated that there is increased expression of iNOS in isolated cultured SGCs in response to CGRP and this is mediated by activation of the MAPKs ERK1/2, JNK and p38 [22]. In the present study, we extended the findings to provide evidence that co-incubation with U0126 or BoNT-A show the same expression of iNOS as seen in fresh specimens; this means that these treatments reduced the organ culture induced increase in iNOS expression.

There is IL-1β immunoreactivity expressed in many neurons in fresh TG. Co-incubation with saline did not change this pattern of expression, however, the MEK1/2 inhibitor U0126 and BoNT-A decreased the expression of IL-1β. There is increasing evidence that proinflammatory cytokines (IL-1 β, IL-6 and TNF-α) either are synthesized in the central (CNS) or in the peripheral nervous system (PNS) by resident cells, or imported by immune blood cells. These are involved in several pathophysiological functions, including an unexpected impact on synaptic transmission and neuronal excitability. Targeting these cytokines, and related signalling molecules, is considered a novel option for the development of therapies in various CNS or PNS disorders associated with an inflammatory component [23].

BoNTs is known to inhibit the release of excitatory neurotransmitters from both motor and sensory neurons by preventing vesicle fusion to cell membrane [24]. Under pathophysiological conditions, BoNTs prevent neurotransmission from almost every type of neuron suggesting common targets present in the majority of neurons. BoNTs specifically and exclusively attack presynaptic nerve terminals. The fusion of a secretory vesicle with the cell membrane is a highly regulated process with the formation of t-SNARE complex playing a pivotal role. The light chain of BoNTs acts to cleave SNAP-25, which inhibits synaptic exocytosis, and therefore, disables neural transmission [25]. We report that the level of expression of SNAP-25 increased after incubation with BoNT-A, but also after NaCl and U0126 incubation. This indicates accumulation of SNAP-25 in the cytosol of the neurons, as a suggestion regarding BoNT-A incubated TG, from cleavage of SNAP-25 inhibiting exocytosis. However, other mechanisms may be involved when it comes to TG incubated with NaCl or U0126.

Synaptic vesicles (SVs), present in all types of neurons [26, 27], are secretory organelles of presynaptic nerve terminals that accumulate high quantities of neurotransmitters and secrete them by fusion with the presynaptic plasma membrane. BoNT-A may enter neurons by binding to synaptic vesicle protein SV2 (isoforms A, B or/and C) [18]. The details of the BoNT-A binding to SV2 has been clarified in hippocampus from knockout mice, demonstrating that those that lacked the isoforms displayed reduced sensitivity to BoNT-A. It was concluded that SV2 acts as the protein receptor for BoNT-A [19]. We showed that the SV2-A isoform is expressed in the SGCs of rat TG, but not in the neurons. This would point towards these cells as a possible site for a putative action of BoNT-A.

The SGCs surround the TG neurons like a pearl necklace, outnumber the neurons in a 10:1 manner, and are joined by gap junctions [20, 28]. The space between the SGCs and the neurons is 20 nm, which allows for an effective control of the extracellular environment by both neurons and SGCs [20]. The role of the SGCs in the function of the trigeminovascular system is poorly known and has by and large received little attention over the years [20]. In previous work, we observed that the SGCs stored CGRP receptor components (RAMP1, CLR) [18]. In the present study we demonstrate the presence of the inflammation marker iNOS. Vast evidence exists for interaction between the SGCs and the TG neurons. Hence these may be activated via peripheral nociceptors such as those within the dura mater (20, 21) and from the temporomandibular joint (TMJ) [23, 29], as well as from central connections in the trigeminal nucleus caudalis (TNC) [13, 30].

The organ culture method has been used in various reports from our laboratory [14–16]. It has been shown that the method could be used to investigate inflammatory events and neuropeptide expression in the TG. In the present study we show that 24 hours of incubation results in condensed nuclear chromatin and compacted cytoplasm in neurons, and enlarged rounded SGCs with highly condensed nuclear chromatin. Moreover, in groups treated with U0126 or BoNT-A, the neurons were often found in a vacuole indicating cell shrinkage and SGC detachment. The impact of these findings in correlation with the expressional alterations of CGRP, iNOS, IL-1 β, SNAP-25 and SV2-4 can only be speculated upon. Pannese (2010) highlighted the striking morphological changes that SGCs undergo after nerve injury, which includes hypertrophy and formation of bridges with other SGCs, which contain numerous newly formed gap junctions [31]. Obviously, SGCs can sense injury-related changes in the neurons, which might have influenced some of the expressional alterations demonstrated here.

Recently, we asked if a chronic inflammation elicited with injection into the TMJ of CFA [32] or CGRP [33], of CFA on the dura mater (Lukács M, Haanes KA, Majláth Z, Tajti J, Vécsei L, Warfvinge K, Edvinsson L. Exposure of the rat dura mater to inflammatory soup or Complete Freund’s Adjuvant induces an inflammatory response in the rat trigeminal ganglion.) might result in altered expression of various markers in the TG. We feel it is of particular importance to maintain the cellular organization because activation of trigeminal neurons leads to changes in adjacent glia that may involve communication through gap junctions and paracrine signaling [17]. The animals were followed for a week and during this time there was a successive increase in inflammation mediators in SGCs and neurons. Interestingly, this enhanced expression could be modified by mitogen-activated protein kinase (MAPK) inhibition *in vivo* or modified by interacting with the glutamatergic system. In the present method of organ culture we found activation of some of these molecules. The MAPK inhibitor U0126 could modify the expression of CGRP and IL-β. On the other hand the inhibition of a single molecule such as CGRP receptor telcagepant did not change the expression (data not shown). Interestingly, BoNT-A had the same effect as for U0126.

## Conclusion

We hypothesize that chronic migraine might be associated with some degree of inflammation in the TG that could involve both neurons and SGCs. It is a clinically well recognized that treatment with corticosteroids will reduce the symptoms of chronic migraine; however this remedy is associated with long-term side effects. Understanding the mechanisms involved in the expressional alterations in the trigeminal system may suggest novel ways to modify the changes and indicate novel therapeutics. The results of the present work illustrate one way by which BoNT-A could modify these expressional alterations in the sensory TG.

## Abbreviations

TG: Trigeminal ganglion
SGC: Satellite glial cells, PBS, Phosphate buffered saline
BSA: Bovine serum albumin
MAPK: Mitogen-activated protein kinases
CGRP: Calcitonin gene-related peptide
U0126: Mitogen activated kinase kinase (EK1/2) inhibitor
BoNT-A: Onabotulinumtoxin type A
iNOS: Inducible nitric oxide synthase
IL-1β: Interleukin 1β
SNAP-25: Synaptosome-associated protein of 25 kDa
SV2-A: Synaptic vesicle protein 2
